# The Effect of Verbal Task Instruction on Spatial-Numerical Associations of Response Codes Effect Coding of Spatial-Numerical Associations: Evidence From Event-Related Potential

**DOI:** 10.3389/fnins.2022.648095

**Published:** 2022-02-15

**Authors:** Yun Pan, Zhiwei Zhang, Wei Li, Xiaoxiao Zhao

**Affiliations:** Key Laboratory of Basic Psychological and Cognitive Neuroscience, School of Psychology, Guizhou Normal University, Guiyang, China

**Keywords:** SNARC effect, verbal-spatial instruction, coding, SOAs, LRP

## Abstract

The spatial-numerical associations of response codes (SNARC) effect reveals that individuals can represent numbers spatially. In this study, event-related potential (ERP) technology was used to probe the effect of verbal-spatial task instructions on spatial-numerical association coding by using digit parity and magnitude judgment tasks, with the numbers 1–9 (except 5) and Chinese word labels (“left” and “right”) as experimental materials. The behavioral results of Experiment 1 showed that the SNARC effect was mainly based on verbal-spatial coding and appeared when the stimulus onset asynchrony (SOA) between the presentation of the verbal labels and the target digit was 0 ms. ERP results did not reveal any significant SNARC-related effects in either the N1 or P3 components. The behavioral results of Experiment 2 again showed that the SNARC effect was dominated by verbal-spatial coding. ERP results showed that significant effects related to verbal-spatial coding were found in both the early positive deflection of the stimulus-locked lateralized readiness potential (S-LRP) and the latency of the response-locked LRP (R-LRP). Hence, in this study, the nature of the spatial coding of the digit magnitudes was influenced by the processing of the word labels and affected both the response selection and response preparation stages.

## Introduction

Spatial-numerical associations are a hot topic in the field of numerical cognition. As one of the most important pieces of evidence for such associations, the spatial-numerical association of response codes (SNARC) has been widely studied. SNARC refers to the phenomenon that participants respond more quickly to small numbers (e.g., 1, 2) with their left hands in numerical magnitude comparison or parity judgment tasks, whereas for large numbers (e.g., 8, 9) they respond faster with their right hands (Dehaene et al., 1993). Since Dehaene et al. (1993) first discovered the SNARC effect, many studies have explored it further. Researchers have verified the SNARC effect for symbolic digits, such as English and German numeral symbols (Calabria and Rossetti, 2005; Nuerk et al., 2005); non-numerical symbols, such as dot matrices (Kadosh et al., 2011); stimulus channels, such as auditory ones (Weis et al., 2016a,b); response modes, such as binocular responses (Hesse and Bremmer, 2017) and one-handed responses (Fischer and Shaki, 2017); and perceptual properties, such as brightness (Ren et al., 2011; Fumarola et al., 2014).

Two main explanations for the SNARC effect itself involve considerations of either the mental number line or the influence of polarity correspondence. The mental number line account assumes that numbers are represented in space in a left-right order from small to large. Therefore, the left hand responds more quickly to small numbers and the right hand to large numbers. A study in patients with brain injuries provided key evidence supporting the mental number line hypothesis (Zorzi et al., 2002). Namely, patients with left neglect were asked to perform a midpoint task involving the numbers 1–9. Results showed that participants shifted their subjective midpoints rightward (e.g., to the number 7) which was highly consistent with a neglect of the left side of spatially represented mental number line. On the other hand, the polarity correspondence account for the SNARC effect holds that numbers are not arranged from left to right, but are related to space through other more conceptual means (Proctor and Cho, 2006). For example, regarding magnitude, the small end of the dimension can be conceptually viewed as having a negative polarity and the large end as having a positive polarity. Spatially, in the horizontal direction, left is considered the negative pole and right to be the positive pole. On this view, the SNARC effect is not caused by a spatial representation of numbers from left to right, but is due to a conceptual correspondence between polarities. One other account that is related to polarity correspondence theory is based on the dual-route model of Gevers et al., 2006. In addition, some researchers have attempted to explain the SNARC effect from the perspective of ordered representations in working memory (van Dijck and Fias, 2011; Ginsburg et al., 2014; Ginsburg and Gevers, 2015; Wang et al., 2018). In general, the mental number line account has been viewed as invoking a visuospatial coding of number magnitude, and the polarity correspondence and dual-route models as invoking a verbal-spatial coding (Gevers et al., 2010).

To verify the nature of the coding underlying the SNARC effect, Gevers et al. (2010) designed four experiments. The first two experiments indicated that either visuospatial coding (by employing the typical left-right manual responding) or verbal-spatial coding (by employing “Left”-“Right” vocal responding) can serve to produce the SNARC effect. Their latter two experiments directly compared the two types of coding (using the words “Left” and “Right” as response labels that were positioned either congruently or incongruently with the spatial location they refer to) and found that in both a parity and a magnitude judgment task, respectively, the SNARC effect was dominated by verbal-spatial coding. However, one important potential confound associated with those latter two experiments was that participants were always instructed to respond according to the identities of the verbal labels (regardless of their spatial positions) when making their judgments. Therefore, the conclusion that verbal-spatial is the main encoding method under such conditions could be regarded as being somewhat controversial (Georges et al., 2015) given that such an instructional context might then serve to induce verbal coding. To explore this issue further, Georges et al. (2015) revised the fourth experiment of Gevers et al. (2010), such that participants were given both verbal and visuospatial task instructions regarding how to respond. In this study, verbal-spatial was shown to be the main encoding method for the verbal-based response instructions, whereas for visuospatial response instructions, both verbal-spatial and visuospatial coding seem to co-exist. Hence, such findings indicate that verbal-spatial coding does not always dominate the SNARC effect, and that visuospatial coding can also play a role. Note, as well, that in Georges et al. (2015), verbal-spatial encoding occurred throughout even though a magnitude judgment task was used (which Gevers et al., 2010, had suggested might itself induce a bias toward visuospatial coding).

It has been found that if two stimuli appear at different times, the first stimulus will affect the processing of the second stimulus depending on the length of the stimulus onset asynchrony (SOA; Pashler, 1994a). The shorter the SOA, the longer the response time to the second stimulus. This phenomenon is called the psychological refractory period (Pashler et al., 2008) with one study finding that the prolongation of response time to the second stimulus likely occurs in the response selection stage (Luck, 1998). In the literature, a few studies have explored the effect of SOAs on the SNARC effect to varying degrees (Daar and Pratt, 2008; Dodd et al., 2008; Dodd, 2011; Schuller et al., 2015). In the study of Gevers et al. (2010), their third experiment also systematically examined whether the length of the SOA between the initial presentation of the “Left” and “Right” verbal response labels and the target number has an effect on the coding mode underlying the SNARC effect (i.e., by potentially allowing the “verbal-spatial and visuospatial components to exert their effects in different time windows” p. 185). In a paradigm such as that employed by Gevers et al. (2010), the identity of the word labels need to be processed before the target number can be responded to. Hence, when SOAs are employed, the time taken to respond to the target number should certainly depend on the time that is available to initially process the word labels. In this regard, an overall main effect of SOA was indeed observed by Gevers et al. (2010) such that times to respond were shorter for longer SOAs (and longer for shorter SOAs). Of greater concern, however, was whether the nature of the verbal-spatial and visuospatial coding of the target number magnitude could be shown to depend on the length of the SOA. Given the lack of an interaction of SOA with any the other factors in the design, this did not seem to be the case in Gevers et al. (2010). That is, a pattern of responding consistent with verbal-spatial coding occurred in their third experiment that did not differ significantly across SOAs. So far, however, such a conclusion rests on the results of only that one particular experiment.

In a related vein, previous studies using electrophysiological techniques have focused on the occurrence time of the SNARC effect. One study analyzed event-related potential (ERP) components time-locked to either the stimulus or the response and found that more robust SNARC effects occurred in the response-locked component. Moreover, further analysis determined that the SNARC effect occurred in the response selection stage rather than the response execution stage (Keus et al., 2005; see also Gevers et al., 2006). Another study on the shift of spatial attention caused by numbers found that the spatial representation of numbers was not related to early sensory attention, but related to the stimulus classification stage (Salillas et al., 2008). On the other hand, subsequent research has indeed also demonstrated the presence of SNARC effects in the amplitudes of both earlier sensory-related components such as the N1 and N2 and more central classification-related components such as the P3 (Gut et al., 2012; see also Pinto et al., 2018). Hence, from such previous ERP work, no consistent conclusion regarding the timing of the SNARC effect can be reached. Note that previous work has also highlighted the fact that spatial-numerical associations could be regarded as automatic activation phenomena (Fischer, 2003; Hubbard et al., 2005) and have come to different conclusions on the dominant hemisphere of digit processing, indicating that the relative contributions of the left and right hemispheres cannot be ignored in the study of spatial representation of numbers (Dormal et al., 2012; Gut et al., 2012; Nikolaev et al., 2020).

As mentioned, associations involving either verbal-spatial or visuospatial coding mechanisms can serve to induce SNARC effects. However, most existing research utilizes tasks that involve lateralized visuospatial responding (Cipora et al., 2019; Pellegrino et al., 2019; Uccula et al., 2020) with only a small few utilizing verbal-spatial responding to explore this effect. Namely, classic SNARC experiments typically map responses to the left and right hands, and only in the balance of the experimental design is each number mapped to the two hands. For example, in the first half of the experiment, the left hand might be used to respond to small numbers and the right hand to large numbers with the left hand responding to large numbers and the right hand to small numbers in the second half of the experiment. In order to separate verbal-spatial coding from visuospatial coding, Gevers et al. (2010) designed their third and fourth experiments to present word labels on each side of the target number and switched their positions randomly across trials. That is, in half of the trials, the word label “Left” appeared to the left of the target number and the word label “Right” appeared to the right of the target number (i.e., the words were congruent with their spatial position) and in the other half of the trials, this positioning was reversed (i.e., the words were incongruent with their spatial position). This allowed for a separation of the inherent verbal-spatial and visuospatial relationship between the magnitude of the target number and the responses. If the magnitude of the number is only represented visuospatially, the SNARC effect will reflect the connection between the magnitude of the number and the spatial position of the response. However, when spatially relevant word labels are added to both sides of the target number, verbal-spatial representation of number magnitude will be reflected in associations of number magnitude with the verbal labels.

In summary, there are very few experiments that have studied the role of verbal-spatial coding on the elicitation of the SNARC effect using the methods introduced by Gevers et al. (2010) and only one experiment that has looked at the effect of manipulating the SOA between the presentation of the verbal labels and the target digits. Hence, a re-examination of that Gevers et al. (2010) paradigm will be undertaken. Because different task components may be affected at different time points after stimulus presentation by conflicts elicited verbal-spatial coding of digit magnitudes and also because the influence of such coding might very well depend on the timing between the encoding of the verbal labels and the target digits, ERP technology was used to directly compare the effect of the relative strength of visuospatial and verbal-spatial coding on the neural underpinnings of the SNARC effect across changes in the length of the SOA. For this, the N1 component corresponding to early sensory attention and the P3 component corresponding to stimulus-response selection and classification were selected for study (Hillyard et al., 1998; Conroy and Polich, 2007). In a second follow-up experiment, the ERP component most closely related to response preparation, namely, the lateralized readiness potential (LRP; Smulders and Miller, 2011) was selected for study for this same task (using the SOA for which the clearest SNARC effects occurred in the first experiment).

## Experiment 1

### Materials and Methods

#### Participants

Twenty-six college students from a university in Guizhou participated in the electroencephalogram (EEG) experiment (14 females, aged 21.78 ± 1.77 years old). All subjects were right-handed, with normal vision or corrected vision, no color blindness or color weakness, no mental history, and good health. They had not participated in similar experiments. The subjects provided written informed consent before the experiment and received remuneration after the experiment. As in previous similar studies (van Dijck et al., 2014; Pinto et al., 2018), G*Power 3.1 software was used to determine that for a power of 0.85, an alpha of 0.05, and an effect size of 0.25, 12 subjects were required. This experiment was approved by the Ethics Committee.

#### Design

As in the third experiment of Gevers et al. (2010), the present experiment had a 4 (SOAs: 0, 200, 800, and 1,200 ms) × 2 (word congruency: the word label “左,” meaning left, appears on the left of the target number, and the word label “右,” meaning right, appears on the right of the target number for word congruent; the word label “右” appears on the left of the target number, and the word label “左” appears on the right of the target number for word incongruent) × 2 (physical congruency: left-hand responses to numbers less than 5 and right-hand responses to numbers greater than 5 is physically congruent; right-hand response to numbers less than 5 and left-hand responses to numbers greater than 5 is physically incongruent) design.

**FIGURE 1:**
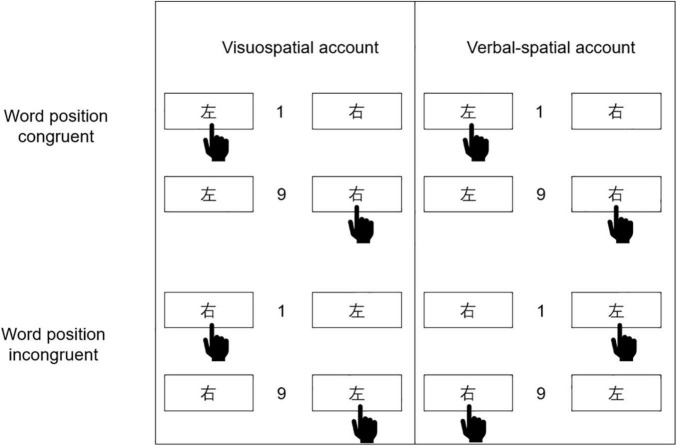
Visuospatial and verbal-spatial account of SNARC effect.

According to Gevers et al. (2010), word congruency refers to whether word labels are in a congruent position (“Left-左” and “Right-右” arranged from left to right) or incongruent position (“Right-右” and “Left-左” arranged from left to right). The position of the hand gesture in Figure 1 represents how the corresponding encoding method prefers to react. According to the visuospatial coding account (see the left side of Figure 1), regardless of the word label, responding is facilitated when the visuospatial aspect associated with the magnitude of the number is consistent with the direction of the responding hand, such that the left hand responds faster to smaller numbers, and the right hand responds faster to larger numbers. Hence, it can be expected that if the SNARC effect is coded visuospatially, the normal SNARC effect will occur regardless of whether the words are congruent or incongruent. Such a result would be signaled by the presence of an overall main effect of physical congruency. According to the verbal-spatial coding account (see the right side of Figure 1), regardless of the actual left or right side of the response, responding is facilitated when the verbal-spatial aspect associated with the magnitude of the number is consistent with the spatial meaning conferred by the word label, such that responses to the label “左” (i.e., “Left”) are faster for smaller numbers, and responses to the label “右” (i.e., “Right”) are faster for larger numbers. Therefore, when the word labels are congruent with their actual spatial positions, the left hand should respond faster to smaller numbers and the right to larger numbers (i.e., a normal SNARC effect, as would also be predicted by visuospatial coding). However, when the word labels are incongruent with their actual spatial positions, the right hand should now respond faster to smaller numbers and the left to larger numbers (i.e., a reverse SNARC effect). Such a result would then be signaled by the presence of an interaction between word congruency and physical congruency.

#### Materials and Procedure

**FIGURE 2:**
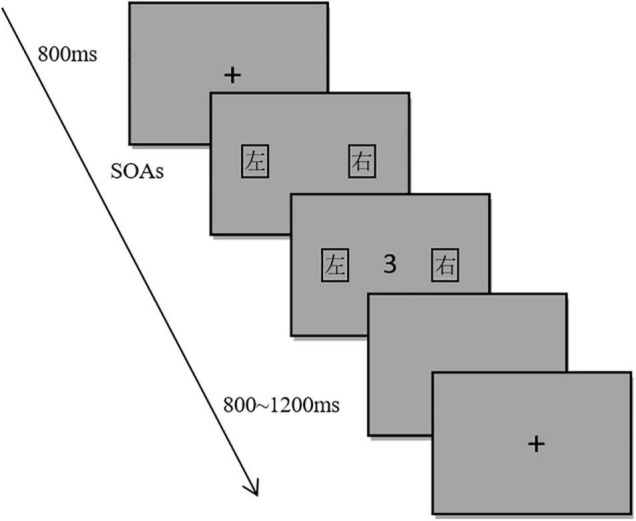
Flow chart of Experimental 1.

The experimental material consisted of two parts: word labels and target numbers. The target numbers were 1–9 (except 5). The number font was Courier New and the size was 1.5° × 2.1°. The font of the word label was Microsoft YaHei, the size is 3° × 3°, and the distance from the number to the word label was 6.5°. The word labels and target numbers were both black, and the background color was grayscale. The experimental program was compiled by MATLAB 2016a. The experimental material was presented on a 21-inch LCD. The screen resolution was 1920 × 1080 and the refresh rate was 100 Hz. The subjects were 75 cm away from the screen, and the fixation point and target number appeared in the center of the screen. In the experiment, the reaction time, accuracy, and EEG data collection were recorded. First, a black fixation spot “+” (1.5° × 1.5°) was displayed in the center of the screen for 800 ms. After the fixation disappeared, word labels appeared on both sides of the screen, followed by the target number (see Figure 2). The interval between the word labels and the target stimulus was randomly set to be either 0, 200, 800, or 1,200 ms. After the subjects completed the response, they were presented with an empty screen of 800–1,200 ms before starting a new trial. The subjects performed a parity judgment task and were randomly divided into two groups. Half of the subjects judged the odd number by pressing the key on the side with the word label “左” and the even number by pressing the key on the side with the word label “右” in the first half of the trials. In the second half of the trials, the odd number was judged using the key on the side with the word label “右” and the even number using the key on the side with the word label “左.” For the other half of the subjects, the order of these mappings was the opposite. The instructions emphasized both speed and accuracy.

The experiment included two blocks of practice trials and eight blocks of formal trials. The practice blocks included 32 trials. The correct rate that the subjects needed to reach was 90% to start the formal trials. If this correct rate is not reached, the practice trials were restarted. For the formal trials, each block consisted of 80 trials. The frequency of each number was balanced, and the order of trials was randomized: 10 times for each number, 40 times for words consistent, 40 times for words inconsistent, and 20 times for each of the four SOAs conditions. There were 640 formal trials and 32 practice trials, yielding a total of 672 trials. After each block, the subjects were given time to rest.

#### Electroencephalogram Recordings and Data Processing

In this study, we used the Neuroscan 4.5 EEG recording system and 64 channels cap to record EEG data. During online recording, the head is taken as a reference, and the middle of the FZ and FPZ is grounded. The left and right electrodes were placed at 1 cm of the left and right eye corners, respectively, and the vertical electrodes were placed 1 cm above and below the orbit of the left eye. The sampling rate was 500 Hz, the filter bandpass was 0.05–100 Hz, and the scalp impedance was less than 5 KΩ. The offline analysis was carried out using EEGLAB and ERPLAB in MATLAB. The filtering band-pass was 50 Hz band stop, and the average value of the bilateral mastoid was used as a reference. The influence of eye movement on EEG data was excluded by independent component analysis (ICA). Before superposition, trials with amplitudes other than ±75 μV were eliminated, and only the correct trials were superimposed. EEG data of 1000 ms were collected. The baseline was 200 ms before the target stimulus was presented (200 ms before the word labels were taken as the baseline when SOA was 200 ms), and the analysis duration was 800 ms after the target presentation. After removing the artifact, the trials were superimposed according to the following 16 treatments: SOA (0, 200, 800, and 1,200 ms) × physical congruency (physically congruent, physically incongruent) × word congruency (word congruent, word incongruent). According to relevant studies, experimental hypotheses (Keus et al., 2005; Gevers et al., 2006; Gut et al., 2012; Pinto et al., 2018), N1 (100 ∼ 200 ms, PO3, PO4, PO5, PO6), and P3 (400 ∼ 550 ms, C1, C2, C3, C4) were selected for statistical analysis. The Greenhouse–Geisser method was used to correct the *p*-values of the main effect and interactions that did not conform to the spherical hypothesis.

### Results

Four subjects were excluded because the artifact rejection rate was higher than 25%, and two subjects were excluded because their error rate was more than 30%.

#### Behavioral Results

SPSS 19 software was used to analyze the reaction time results. Any reaction times longer than three standard deviations were rejected (6%). The analysis involved a 4 (SOAs: 0, 200, 800, and 1,200 ms) × 2 (word congruency: word congruent and word incongruent) × 2 (physical congruency: physically congruent and physically incongruent) repeated measures analysis of variance (ANOVA).

The results showed that the main effect of SOAs was significant, *F*(3,57) = 46.61, *p* < 0.001, η*_*p*_^2^* = 0.71. *Post hoc* comparisons found that reaction time decreased with the increase of SOAs, the reaction time for the SOA of 0 ms (1040 ms) was significantly longer than for the SOA of 200 ms (961 ms), *p* < 0.001; the reaction time for the SOA of 0 ms significantly longer than that for the SOA of 800 ms (903 ms), *p* < 0.001; the reaction time for the SOA of 0 ms significantly longer than that for the SOA of 1200 ms (898 ms), *p* < 0.001; the reaction time for the SOA of 200 ms was significantly longer than for the SOA of 800 ms (*p* < 0.01); the reaction time for the SOA of 200 ms was significantly longer than for the SOA of 1,200 ms (*p* < 0.01); and the reaction time for the SOAs of 800 ms and 1,200 ms were not significantly different. The main effect of word congruency was significant, *F*(1,19) = 12.38, *p* < 0.01, η*_*p*_^2^* = 0.39, the reaction time for word congruent trials (935 ms) was significantly shorter than that for word incongruent trials (966 ms); the main effect of physical congruency was not significant, *F*(1,19) = 0.59, *p* > 0.05; the interaction between word congruency and physical congruency was significant, *F*(1,19) = 8.52, *p* < 0.01, η*_*p*_^2^* = 0.31. No other interactions were significant (all *ps* > 0.05). As mentioned, an interaction between word congruency and physical congruency indicates that the SNARC effect is mainly based on verbal-spatial coding (Gevers et al., 2010). The results for each cell of this design are shown in Table 1.

**TABLE 1:** Response time (ms) of parity judgment task (*M* ± *SD*).

	Word congruent		Word incongruent	
SOAs	Physically congruent	Physically incongruent	Physically congruent	Physically incongruent
0 ms	1037.72 ± 244.70	1021.58 ± 211.36	1069.67 ± 196.83	1032.20 ± 196.83
200 ms	944.20 ± 191.17	953.50 ± 182.16	971.05 ± 190.51	975.48 ± 207.15
800 ms	882.28 ± 213.15	897.97 ± 214.73	920.79 ± 211.01	911.27 ± 215.29
1200 ms	855.85 ± 225.10	885.15 ± 208.28	937.94 ± 253.69	912.07 ± 239.84

**FIGURE 3:**
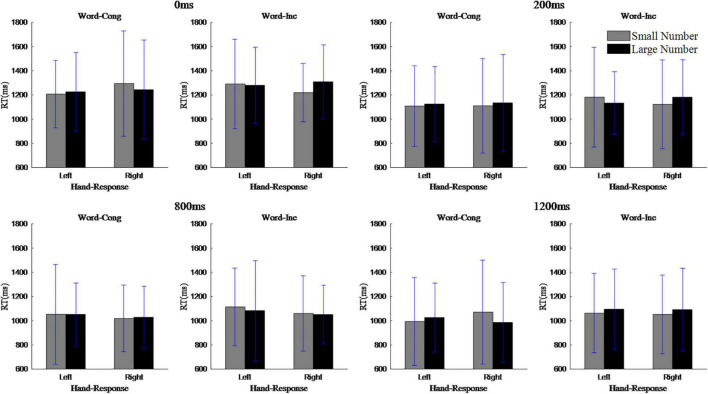
The interaction between magnitude and reaction hand of each stimulus onset asynchrony in the parity judgment task.

To assess the actual SNARC effects, repeated measures ANOVAs were carried out on reaction time that involved a 2 (magnitude: 1, 2, 3, and 4 are small numbers; 6, 7, 8, and 9 are large number) × 2 (response hand: left hand, right hand) design. The results showed that the main effect of the response hand was not significant, *F*(1,19) = 0.46, *p* > 0.05; the main effect of magnitude was not significant, *F*(1,19) = 1.71, *p* > 0.05; and the interaction between response hand and magnitude was not significant, *F*(1,19) = 0.55, *p* > 0.05, indicating that there was no overall SNARC effect. Nonetheless, in the case of word congruent trials, when the SOA was 0 ms, the main effect of response hand was not significant, *F*(1,19) = 1.79, *p* > 0.05; the main effect of magnitude was not significant, *F*(1,19) = 0.71, *p* > 0.05; but the interaction between response hand and magnitude was marginally significant, *F*(1,19) = 4.04, *p* < 0.06, η*_*p*_^2^* = 0.18. Simple effect analysis showed that the reaction time to small numbers (1207 ms) was shorter than to large numbers (1226 ms) when the response hand was left, while the reaction time to small numbers (1295 ms) was longer than the reaction time to large numbers (1244 ms) when the response hand was right (i.e., the normal SNARC effect). In the case of word incongruent trials, when the SOA was 0 ms, the main effect of response hand was not significant, *F*(1,19) = 0.71, *p* > 0.05; the main effect of magnitude was not significant, *F*(1,19) = 4.09, *p* > 0.05; but the interaction between response hand and magnitude was significant, *F*(1,19) = 4.64, *p* < 0.05, η*_*p*_^2^* = 0.20. Simple effect analysis showed that when the response hand was left hand, the reaction time to small numbers (1,291 ms) was longer than that to large numbers (1,280 ms) and when the response hand was right hand, the reaction time to small numbers (1,220 ms) was shorter than that to large numbers (1,309 ms), indicating the presence of reverse SNARC effect. Note that for the other three SOAs, it was never the case that significant interactions between response hand and magnitude were present for both word-congruent and word-incongruent trials. See Figure 3.

#### Event-Related Potential Results

##### N1

**FIGURE 4:**
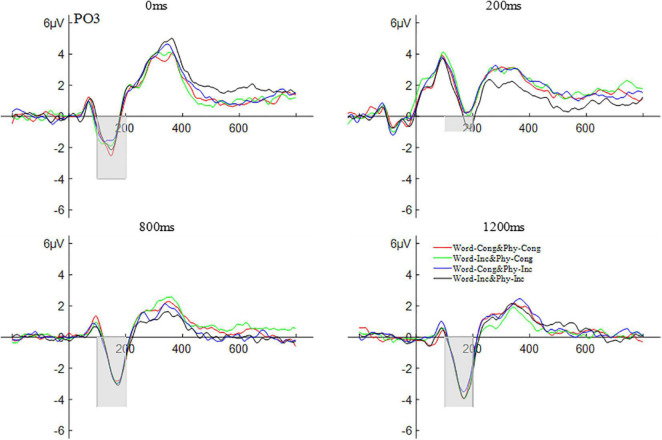
The waveform of N1 word and physical congruency under different stimulus onset asynchrony conditions.

Repeated measures ANOVA was carried out on the amplitude of N1 according to a 2 (brain area: left and right) × 2 (physical congruency: physically congruent, physically incongruent) × 2 (word congruency: word congruent, word incongruent) × 4 (SOAs: 0, 200, 800, and 1,200 ms) design. The results showed that the main effect of SOAs was significant, *F*(3,57) = 19.76, *p* < 0.001,η*_*p*_^2^* = 0.51. *Post hoc* comparisons revealed that the amplitude for the SOA of 0 ms (−5.33 μV) was significantly lower than that for the SOA of 200 ms (−1.19 μV), *p* < 0.001; the amplitude for the SOA of 800 ms (−4.85 μV) was significantly lower than that for the SOA of 200 ms, *p* < 0.001; the amplitude for the SOA of 1200 ms (−5.61 μV) was significantly lower than that for the SOA of 200 ms, *p* < 0.001; with the amplitude differences among other SOAs not being significant, all *ps* > 0.05. No other main effects and interactions were significant (all *ps* > 0.05). The waveform of the N1 component is shown in Figure 4.

##### P3

**FIGURE 5:**
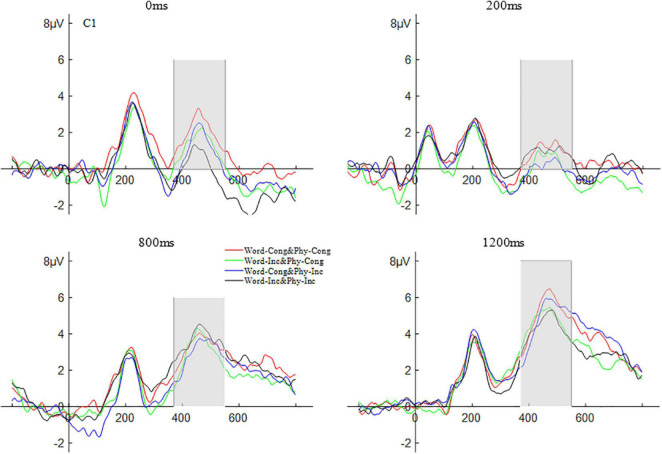
The waveform of P3 word and physical congruency under different stimulus onset asynchrony conditions.

A repeated measures ANOVA was carried out on the amplitude of P3 according to a 2 (brain area: left and right) × 2 (physical congruency: physically congruent, physically incongruent) × 2 (word congruency: word congruent, word incongruent) × 4 (SOAs: 0, 200, 800, and 1,200 ms) design. The results showed that the main effect of SOAs was significant *F*(3,57) = 12.49, *p* < 0.001, η*_*p*_^2^* = 0.39. *Post hoc* comparisons revealed that the amplitude for the SOA of 0 ms (4.24 μV) was significantly less than that for the SOA of 1200 ms (7.48 μV), *p* < 0.01; the amplitude for the SOA of 200 ms (3.55 μV) was significantly smaller than that for the SOA of 800 ms (6.08 μV), *p* < 0.01; the amplitude for the SOA of 200 ms was significantly smaller than that for the SOA of 1200 ms, *p* < 0.001; the amplitude for the SOA of 800 ms was significantly smaller than that for the SOA of 1200 ms, *p* < 0.01; with the amplitude differences among other SOAs not being significant, all *ps* > 0.05. No other main effects and interactions were significant (all *ps* > 0.05). The P3 waveform is shown in Figure 5.

##### Transition Discussion

The main purpose of this experiment is to explore the impact of changing SOAs on the nature of the coding underlying the SNARC effect when responding to verbal labels. The overall analysis of variance revealed a main effect of both SOA and word congruency along with an interaction between word congruency and physical congruency where the latter result signals the occurrence of SNARC effects that relied on verbal-spatial coding. Nonetheless, separate analyses of the SNARC effect itself for word congruent and word incongruent trials at each SOA, indicated that SNARC effects that could be ascribed to verbal-spatial coding occurred mainly when the SOA was 0 ms. Namely, for word congruent trials at the 0 ms SOA, a normal SNARC effect was present, whereas for word incongruent trials at the 0 ms SOA, a reverse SNARC effect was present. Interestingly, such results suggest that the verbal-spatial coding of target digit magnitude was indeed strongest for the case in which the verbal labels were presented simultaneously with the target digit and became less influential as the presentation of the labels and the target digit became more separated in time. Namely, the longer the SOA, the effect of processing the verbal labels on the spatial coding of digit magnitude became more diminished and the less likely it was that a verbal-spatial coding of the magnitude of the target digits occurred.

One caveat, though, is that in the first experiment parity judgments were being made which might still then have resulted in a bias toward verbal-spatial coding. In contrast, the task of judging number magnitude is more likely to involve explicitly arranging numbers from left to right thereby inducing an inherent bias toward visuospatial coding. Hence, in the second experiment, a magnitude judgment task is utilized. As well, given the presence of SNARC effects for both the word congruent and word incongruent trials at the 0 ms SOA only in the first experiment, no further manipulation of SOA is examined in the second experiment.

In the ERP results, neither the N1 component, which marks early attention, nor the P3 component, which is affected by central cognitive and executive functioning, revealed significant effects of either word congruency or physical congruency or an interaction between them, thereby demonstrating that the SNARC effect was did not seem to be evident here at the level of such electrophysiological results. Nonetheless, given the spatial basis of this effect, an examination of its representational basis at the electrophysiological level would likely be more fruitful for potentials that are more closely related to the response preparation processes underlying the actual manual responding itself. Hence, the second experiment examined the Lateralized Readiness Potential (LRP) that is generated right before the individual makes voluntary movements. The LRP is a component that corresponds to response preparation. It refers to the presence of a significant negative potential on the opposite scalp position to the responding hand and is related to the unconscious initiation of the response by the brain before any voluntary movement occurs.

## Experiment 2

### Materials and Methods

#### Participants

Thirty-one college students from a university in Guizhou participated in the EEG experiment (18 female students, aged 22.71 ± 2.08 years old). All subjects were right-handed, with normal vision or corrected vision, no color blindness or color weakness, no mental history, or good health. They had not participated in similar experiments. The subjects signed informed consent before the experiment and received remuneration after the experiment. As in previous studies (van Dijck et al., 2014; Pinto et al., 2018), G*Power 3.1 software was used to calculate the power of 0.85, the alpha set of 0.05 and the effect size of 0.25, which required 26 subjects. This experiment was approved by the ethics committee.

#### Design

The experiment had a 2 (word congruency: the word label “左” (meaning left) appears on the left of the target number and the word label “右” (meaning right) appears on the right of the target number for word congruent; the word label “右” appears on the left of the target number and the word label “左” appears on the right of the target number for word incongruent) × 2 (physical congruency: left-hand responses to numbers less than 5 and right-hand responses to numbers greater than 5 is physically congruent; right-hand responses to numbers less than 5 and left-hand responses to numbers greater than 5 is physically incongruent) design.

The LRP can separate out the reaction-related components from the other components through the averaging method. The basic logic is to subtract the voltage value of the electrode point on the same side as the responding hand from the voltage value of the electrode point on the opposite side of the responding hand and then to average the two subtractions across responses made with each hand. In the resulting LRP waveform, the correct response will show a negative deflection of the unilateral preparation potential component, and the incorrect response will show a positive deflection. In an experiment involving spatial-numerical associations, the direction of the LRP shift induced by different coding methods would be different. Specifically, according to the visuospatial coding account, number magnitude would activate the same spatially congruent response hand under the condition of both word congruency and word incongruency. This would result in a negative deflection of the LRP for physically congruent trials and positive (or less negative) deflection for physically incongruent trials regardless of word congruency or incongruency. According to the verbal-spatial coding account, however, the response hand on the side of the spatially congruent verbal label would be activated. This would result in a negative deflection of the LRP for word congruent trials that are also physically congruent but also for word incongruent trials that are also physically incongruent (because, in both cases, smaller numbers would be responded to using the “Left – 左” verbal label and larger numbers would be responded to using the “Right – 右” verbal label). On the other hand, a positive (or less negative) deflection would be expected for both word congruent but physically incongruent trials or word incongruent but physically congruent trials.

#### Materials and Procedure

**FIGURE 6:**
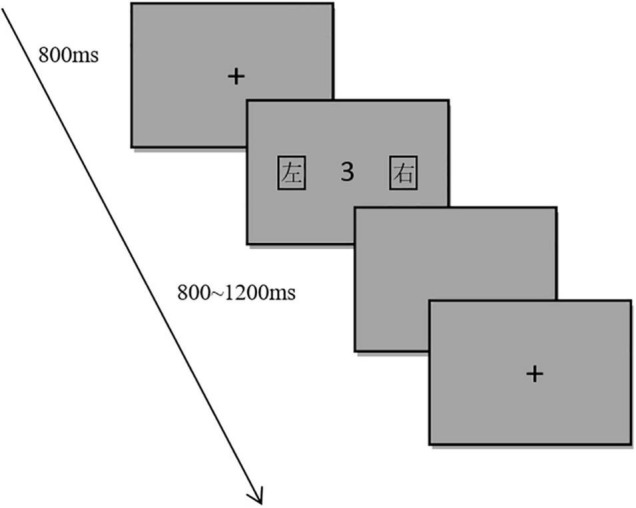
Flow chart of Experimental 2.

The experimental materials were the same as in Experiment 1, as shown in Figure 6.

The experiment includes two blocks of practice trials and eight blocks of formal trials. The practice blocks included 32 trials. The correct rate that the subjects needed to reach was 90% to start the formal trials. If this correct rate is not reached, the practice trials were restarted. For the formal trials, each block consisted of 80 trials. The frequency of each number was balanced, and the order of trials was randomized: 10 times for each number, 40 times for words consistent, and 40 times for words inconsistent. There were 640 formal trials and 32 practice trials, yielding a total of 672 trials. After each block, the subjects were given time to rest.

#### Electroencephalogram Recording and Data Processing

The EEG recording and data processing were the same as in Experiment 1. The recording electrode channels of LRP were C3 and C4, and the calculation formula was LRP = [(C3-C4) right hand + (C4-C3) left hand/2. The stimulus-locked LRP (S-LRP) was taken from 200 ms before stimulation until 800 ms after stimulation, with 200 ms before stimulation as the baseline. The response-locked (R-LRP) was taken from 800 ms before the reaction until 200 ms after the reaction, with 800–600 ms before the reaction as the baseline.

### Results

Four subjects were excluded because the false mark rejection rate was higher than 25%, and one subject was excluded because their error rates were more than 30%. A total of 26 subjects were analyzed.

#### Behavioral Results

SPSS 19 software was used to analyze the results. Any reaction times longer than three standard deviations were rejected (5%). The analysis involved a 2 (word congruency: word congruent and word incongruent) × 2 (physical congruency: physically congruent and physically incongruent) repeated measures analysis of variance (ANOVA). The results show that the main effect of word congruency was significant, *F*(1,25) = 27.33, *p* < 0.001, η*_*p*_^2^* = 0.52, the mean reaction time for word congruent trials (902 ms) was significantly shorter than that for word incongruent trials (933 ms); the main effect of physical congruency was not significant, *F*(1,25) = 0.76, *p* > 0.05; and the interaction between word congruency and physical congruency was significant, *F*(1,25) = 5.36, *p* < 0.05, η*_*p*_^2^* = 0.18. Accordingly, this interaction indicates that the SNARC effect is the result of verbal-spatial coding of numerical magnitude (Gevers et al., 2010). The results for each cell of this design are shown in Table 2.

**TABLE 2:** Response time (ms) of magnitude judgment task (*M* ± *SD*).

	Physically congruent	Physically incongruent
Word congruent	864.75 ± 189.35	941.11 ± 148.45
Word incongruent	965.74 ± 156.68	899.45 ± 184.75

**FIGURE 7:**
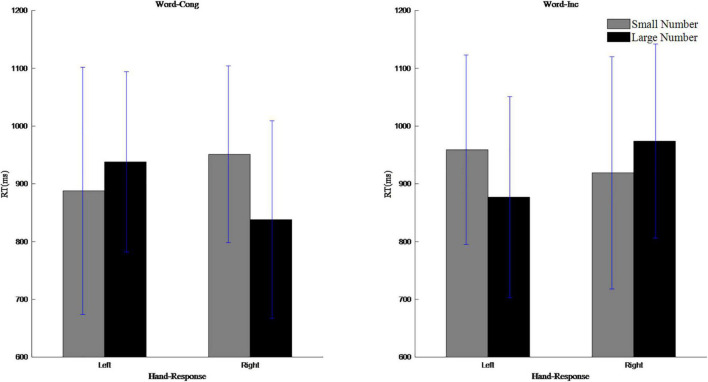
The interaction between magnitude and reaction hand in magnitude judgment task.

To assess the actual SNARC effects, repeated measures ANOVAs were carried out on reaction time that involved a 2 (magnitude: 1, 2, 3, and 4 are small numbers; 6, 7, 8, and 9 are large number) × 2 (response hand: left hand, right hand) design. In the case of word congruent trials, the results showed that the main effect of response hand was not significant, *F*(1,25) = 2.19, *p* > 0.05; the main effect of magnitude was significant, *F*(1,25) = 6.02, *p* < 0.05, η*_*p*_^2^* = 0.19; and the interaction between the response hand and magnitude was significant, *F*(1,25) = 6.02, *p* < 0.05, η*_*p*_^2^* = 0.19. Simple effect analysis showed that when the response hand was left, the reaction time for small numbers (888 ms) was shorter than for large numbers (939 ms), but when the response hand was right, the reaction time for small numbers (951 ms) was longer than for large numbers (839 ms), indicating the occurrence of a normal SNARC effect. In the case of word incongruent trials, the main effect of response hand was significant, *F*(1,25) = 4.80, *p* < 0.05, η*_*p*_^2^* = 0.16; the main effect of magnitude was not significant, *F*(1,25) = 2.47, *p* > 0.05; and the interaction between the response hand and magnitude was significant, *F*(1,25) = 4.80, *p* < 0.05, η*_*p*_^2^* = 0.16. Simple effect analysis showed that when the response hand was left, the reaction time for small numbers (959 ms) was longer than for large numbers (877 ms), but when the response hand was right, the reaction time was shorter for small numbers (920 ms) than for large numbers (975 ms), indicating the occurrence of a reverse SNARC effect. See Figure 7.

#### Event-Related Potential Results

##### Stimulus-Locked Lateralized Readiness Potential

**FIGURE 8:**
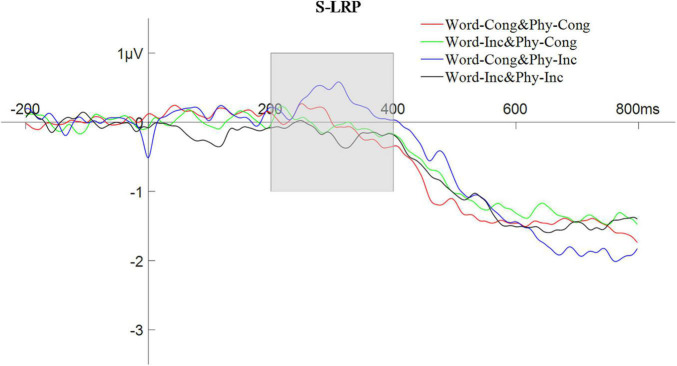
The waveform of stimulus-locked lateralized readiness potential with word and physical congruency.

The peak amplitude of the S-LRP, in the time window of 200–400 ms, was analyzed using a repeated measures ANOVA with a 2 (physical congruency: physically congruent, physically congruent) × 2 (word congruency: word congruent, word incongruent) design. The results showed that the main effect of physical congruency was not significant, *F*(1,25) = 4.18, *p* > 0.05; the main effect of word congruency was significant, *F*(1,25) = 7.98, *p* < 0.01, η*_*p*_^2^* = 0.24; and the interaction between physical congruency and word congruency was also significant, *F*(1,25) = 5.48, *p* < 0.05, η*_*p*_^2^* = 0.18. For an analogous ANOVA involving the S-LRP latency in the time window of 200–600 ms with 30% partial area, the main effect of physical congruency was not significant, *F*(1,25) = 0.02, *p* > 0.05; the main effect of word congruency was not significant, *F*(1,25) = 1.79, *p* > 0.05; and the interaction between physical congruency and word congruency was not significant, *F*(1,25) = 0.12, *p* > 0.05. The waveform of the S-LRP composition is shown in Figure 8.

##### Response-Locked LRP

**FIGURE 9:**
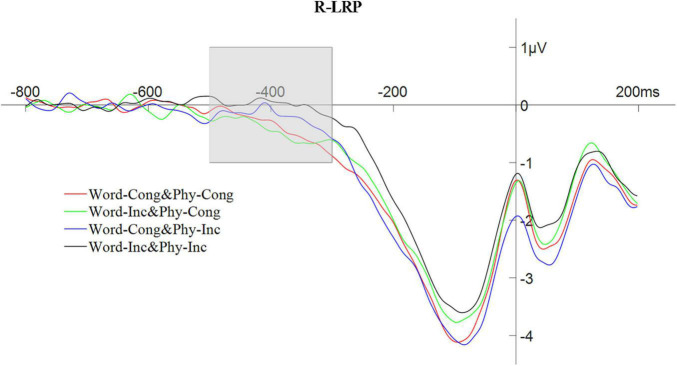
The waveform of response-locked lateralized readiness potential with word and physical congruency.

The peak amplitude of the R-LRP, in the time window of −500 to −300 ms, was also analyzed using a repeated measures ANOVA with a 2 (physical congruency: physically congruent, physically incongruent) × 2 (word congruency: word congruent, word incongruent) design. The results showed that the main effect of physical congruency was not significant, *F*(1,25) = 0.95, *p* > 0.05; the main effect of word congruency was significant, *F*(1,25) = 4.86, *p* < 0.05, η*_*p*_^2^* = 0.16; and the interaction between physical congruency and lexical congruency was not significant, *F*(1,25) = 0.01, *p* > 0.05. For an analogous ANOVA involving the R-LRP latency in the time window of −550 to −50 ms with 30% partial area, the main effect of physical congruency was not significant, *F*(1,25) = 3.85, *p* > 0.05; the main effect of word congruency was not significant, *F*(1,25) = 3.68, *p* > 0.05; but the interaction between physical congruency and word congruency was significant, *F*(1,25) = 4.36, *p* < 0.05, η*_*p*_^2^* = 0.15. The waveform of the R-LRP composition is shown in Figure 9.

## Discussion

In Experiment 1, a parity judgment task was used to explore the encoding mode underlying the spatial coding of numerical magnitude by manipulating the word and physical congruency of the responses across four SOAs. In Experiment 2, the magnitude judgment task was used to explore this same issue. The behavioral results for both experiments were consistent with verbal-spatial coding. Namely, the nature of the spatial-numerical associations that would be expected for such coding would be manifested in terms of normal SNARC effects in the case where the words were displayed congruently with the locations they refer to, and in terms of reverse SNARC effects in the case where the words were displayed incongruently with the locations they refer to – which was indeed observed in the present results. Previous studies have suggested that the coding mode underlying the SNARC effect is mainly verbal-spatial (Gevers et al., 2010). However, some researchers believe that the SNARC effect can be highly dependent on task instructions, and that spatial-numerical associations are not the result of a single processing mechanism. Namely, Georges et al. (2015) proved strong evidence that verbal-spatial coding is the main encoding mode of the SNARC effect when instructions emphasize verbal-spatial responding, but that visuospatial coding also plays a role when instructions emphasize visuospatial responding.

The present experimental results also show that reaction time decreases with the increase in SOAs, which is consistent with the results of dual-task paradigms. As well, only when SOA was 0 ms were significant SNARC effects found for both word-congruent and word-incongruent trials, with no such effects being observed at the other three SOAs. Note that such a result is inconsistent with previous results (i.e., Gevers et al., 2010) which led to the conclusion by those researchers that SNARC effects do not depend on SOA. Hence, unlike that previous study, the present results do provide some evidence that the verbal-spatial coding of digit magnitude is more likely the more simultaneous the processing of the verbal labels and the target digit (allowing the processing of the former to affect the magnitude coding of the latter). As well, the notion of a central bottleneck, (in which the processing of one of two closely presented stimuli needs to wait until the processing of the other has been completed; Pashler, 1994b) could also help to explain why such SNARC effects appeared for the 0 ms SOA but not the other three longer ones. Namely, research has shown that SNARC effects can depend on how efficient a trial can be processed (Fischer and Shaki, 2014; Cipora et al., 2019) with stronger SNARC effects typically being associated with longer reaction times. Hence, the need to process both the word labels and the target digits in very close temporal proximity at the 0 ms SOA enhanced the difficulty of the task which then could have increased the likelihood of observing SNARC effects. Finally, note that all of the digits from 1 to 9 (except 5) were used in the present experiment, whereas only the digits 1, 2, 8, and 9 were utilized in the Gevers et al. (2010) third experiment. Because SNARC effects are typically enhanced for more extreme digits, this point might also have played a role in serving to increase the likelihood that SNARC effects would occur at SOAs longer than 0 ms in Gevers et al. (2010).

There has been no clear and unified conclusion regarding the stages of processing that can be influenced by SNARC-related conflict between the spatial coding of numerical magnitude and the spatial characteristics of the responses (i.e., early sensory stages and/or later response stages). In this vein, the results of the present Experiment 1 did not provide evidence for either a main effect physical congruency or interaction between physical congruency and word congruency in the N1 and P3 components, indicating that neither visuospatial not verbal-spatial coding of digit magnitude seem to be having an electrophysiological effect at either that early sensory or intermediate stimulus classification levels. In Experiment 1, differences in overall N1 were found across SOAs that essentially were due to differences between the 200 ms SOA (for which a slightly earlier baseline was used) and the other SOAs. For the P3 component, however, there was also some evidence that overall P3 amplitude tended to increase with increases in the SOA (i.e., that target digit classification was affected by the overlap, or lack thereof, between the processing of the word labels and target number).

In Experiment 2, there was an interaction of word and physical congruency on LRP amplitude that appeared in the early stages of the stimulus-locked LRP, indicating that verbal-spatial coding affects strength of the activation of the response during the response selection stage occurring prior to the actual preparation and execution of the response (as Keus et al., 2005, also found in their study of the normal SNARC effect). Such a result is consistent with the Dehaene et al. (1998) study of unconscious perception which found that that the incongruence between the magnitude of a priming stimulus and a target stimulus induced an early reverse shift in the stimulus-locked LRP (Dehaene et al., 1998). Note that such a shift could then be presumed to be induced by the automatic processing of the priming stimuli. Therefore, the current s-LRP results are in line with the notion that spatial-numerical associations do not need attention and represent an automatic activation phenomenon (Fischer, 2003; Hubbard et al., 2005). Furthermore, in Experiment 2, an interaction between word and physical congruency was found in the latency of the response-locked LRP [in contrast to the lack of normal SNARC-related effects found in the r-LRP by both Keus et al. (2005) and Gevers et al. (2006)], indicating that verbal-spatial coding indeed also seems to affect later stages related to the motor preparation of the response.

However, there are some limitations in this study. First, the reading habits of the subjects in this study were from left to right, and the extent to which coding mode is affected by the reading habits is unknown. Second, the number of subjects in this study is small, and large sample studies are needed to further verify the findings. Finally, the number of trials for each condition in Experiment 1 was not that large given the need to include the four SOAs (although increasing the number of trials would have led to excessive fatigue). In the future research, the experimental procedures should be further optimized and subjects with different cultural reading habits selected in order to verify the results.

## Conclusion

This study involves adult subjects, who were asked to perform judgments of either the numerical parity or magnitude of single digits by making manual responses to “Left” and “Right” verbal labels, and used event-related potential technology to assess the nature of the underlying spatial coding and time of occurrence of the corresponding SNARC effects. The results of Experiment 1 provided evidence that that the SNARC effect is based on verbal-spatial coding of digit magnitude that also depended on the SOA between the display of the verbal response label options and the digits themselves. The results of Experiment 2 provided more evidence that that the SNARC effect is based on verbal-spatial coding, and that such coding can affect the response-related stages of processing.

## Data Availability Statement

The raw data supporting the conclusions of this article will be made available by the authors, without undue reservation.

## Ethics Statement

The studies involving human participants were reviewed and approved by the Ethics Committee of School of Psychology, Guizhou Normal University. The patients/participants provided their written informed consent to participate in this study.

## Author Contributions

ZZ and YP: conception and design of the study, and drafting the manuscript. ZZ and XZ: acquisition of data. ZZ and WL: data analysis and revise the manuscript. All authors contributed to the article and approved the submitted version.

## Conflict of Interest

The authors declare that the research was conducted in the absence of any commercial or financial relationships that could be construed as a potential conflict of interest.

## Publisher’s Note

All claims expressed in this article are solely those of the authors and do not necessarily represent those of their affiliated organizations, or those of the publisher, the editors and the reviewers. Any product that may be evaluated in this article, or claim that may be made by its manufacturer, is not guaranteed or endorsed by the publisher.
